# Experimental Validation of MHC Class I and II Peptide-Based Potential Vaccine Candidates for Human Papilloma Virus Using Sprague-Dawly Models

**DOI:** 10.3390/molecules28041687

**Published:** 2023-02-10

**Authors:** Mehreen Ismail, Baogang Bai, Jinlei Guo, Yuhui Bai, Zureesha Sajid, Syed Aun Muhammad, Rehan Sadiq Shaikh

**Affiliations:** 1 Institute of Molecular Biology and Biotechnology, Bahauddin Zakariya University, Multan 60800, Pakistan; 2 School of Information and Technology, Wenzhou Business College, Wenzhou 325015, China; 3 Engineering Research Center of Intelligent Medicine, Wenzhou 325000, China; 4 The 1st School of Medical, School of Information and Engineering, The 1st Affiliated Hospital of Wenzhou Medical University, Wenzhou 325015, China; 5 School of Medical Engineering, Sanquan College of Xinxiang Medical University, Xinxiang 453513, China; 6 Department of Computer Science and Engineering, Southern University of Science and Technology, Shenzhen 518055, China; 7 Centre for Applied Molecular Biology, University of the Punjab, Lahore 54000, Pakistan

**Keywords:** HPV, epitope prediction, peptides vaccine, animal studies, immunoassays

## Abstract

Human papilloma virus (HPV) causes cervical and many other cancers. Recent trend in vaccine design is shifted toward epitope-based developments that are more specific, safe, and easy to produce. In this study, we predicted eight immunogenic peptides of CD4+ and CD8+ T-lymphocytes (MHC class I and II as M1 and M2) including early proteins (E2 and E6), major (L1) and minor capsid protein (L2). Male and female Sprague Dawly rats in groups were immunized with each synthetic peptide. L1M1, L1M2, L2M1, and L2M2 induced significant immunogenic response compared to E2M1, E2M2, E6M1 and E6M2. We observed optimal titer of IgG antibodies (>1.25 g/L), interferon-γ (>64 ng/L), and granzyme-B (>40 pg/mL) compared to control at second booster dose (240 µg/500 µL). The induction of peptide-specific IgG antibodies in immunized rats indicates the T-cell dependent B-lymphocyte activation. A substantial CD4+ and CD8+ cell count was observed at 240 µg/500 µL. In male and female rats, CD8+ cell count for L1 and L2 peptide is 3000 and 3118, and CD4+ is 3369 and 3484 respectively compared to control. In conclusion, we demonstrated that L1M1, L1M2, L2M1, L2M2 are likely to contain potential epitopes for induction of immune responses supporting the feasibility of peptide-based vaccine development for HPV.

## 1. Introduction

Human Papilloma viruses (HPV) are responsible for substantial morbidity and mortality worldwide. HPV is affecting a large set of population in the world, about 311,000 deaths occurred around the globe in 2018 [1]. HPVs are characterized by diversity and complexity, presenting a wide spectrum of clinical forms in humans ranging from self-healing cutaneous lesions to fatal cervical cancers. HPV is also responsible for penile, anal, vulvar and vaginal, oral, laryngeal, and esophageal cancers, but the evidences are not as strong as for cervical cancer [2]. To manage such issues, two FDA (Food and Drug Administration) approved vaccines, gardasil and cervarix, are being used against HPV6, 11, 16, and 18 variants [3]. However, these vaccines are ineffective in sexually active women and do not help with preexisting lesions. They are also not recommended for pregnant women. Cost effectiveness and toxicity are other limitations of these vaccines, e.g., gardasil is linked with Guillain-Barré syndrome (GBS), a rare disorder of the nervous system [4]. Therefore, more safer and effective vaccines are required for a protective immune response. In this study, we predicted and validated potential vaccine candidates against high-risk variants of HPV to combat its drastic effects in the population.

The HPV is a double-stranded DNA virus and it is 50–60 nm in diameter. It contains 72 capsomeres on its surface [5]. This DNA, consisting of 7200–8000 base pairs, represents 10–13% of the virion mass and is enveloped inside capsid proteins [6]. Almost 440 variants of HPV are categorized as high and low risk strains. Among them, HPV16, 18, 31, 33, 35, 39, 45, 51, 52, 56, 58, and 59 are considered high risk variants responsible for infections and different types of cancers [7]. It has been observed that HPV genomes are characterized into early, late, and regulatory regions. Early regions constitute 50% of the viral genome containing E1, E2, E4, E6, and E7 segments. However, the late region contains L1 and L2 genes constituting 40% of genome, leaving 10 percent of regulatory regions [8]. Some of these segments are antigenic and activate the immune system and could be used as potential vaccine candidates. Among HPV proteins, capsid proteins L1 and L2 and regulatory proteins E2 and E6 are comparatively conserved proteins, are able to trigger specific immune responses, and immunization with these proteins confers protection against HPV infection in experimental animal models [9]. Antigen identification is considered as a significant barrier in vaccine design, as this is usually achieved through time-consuming and labor-intensive in vitro and in vivo experiments [10]. Reverse vaccinology is the radical change in vaccine research. The reverse vaccinology approach is the discovery of new antigens through whole genome sequencing following laboratory-based analysis to find efficacy of these antigens to induce protective immunity [11]. Genome wide studies and reverse vaccinology have helped health professionals and scientists to modify prophylactic and therapeutic strategies against a variety of infectious diseases. The availability of genomic and proteomic information now enabled researchers to explore the proteomic segments of these virulent strains without culturing them in labs to save time and resources. These information support to find new antigens against HPV and other infectious agents [12]. Efforts have focused on developing new strategies for rational and fast antigen identification among a number of viral proteins. Furthermore, recent reports support that epitope-based vaccines appear to be capable of inducing more potent responses than whole protein vaccines [13]. T-cell epitope prediction of protein sequences by bioinformatics analysis has been proposed as a promising strategy for vaccine development and an increasing number of tools have been developed, based on different algorithms and methods [14]. T-cells from genetically distinct populations would recognize and respond to a single peptide epitope, underline the need of identifying one or more epitope(s) that bind to multiple HLA (Human leukocyte antigen) alleles and cover close to 100% of the genetically diverse human population. Multi epitope-based vaccines are designed to generate a diverse immune response, to incorporate antigens, and to reduce limitations due to MHC (Major Histocompatibility Complex) restriction to a single entity. The effectiveness of a vaccine depends on its capacity to ensure long-lasting cell-mediated immunity. A murine model is comparable to self-controlled oligosymptomatic cases and therefore is useful for the study of the protective immune response. Several reports demonstrate that different viral antigens elicit desired immune responses capable to sustain protection against experimental challenges [15]. In this study, we used an immunoinformatics approach to identify potential T-cell epitopes of HPV (L1, L2, E2, and E6) and experimentally verified the efficacy of these peptides using Sprague Dawly rats by analyzing peptide-specific proliferative responses and cytokine production of CD8+ and CD4+ T-cells.

## 2. Results

### 2.1. Prediction of T-Cell Epitopes

**Figure 1:**
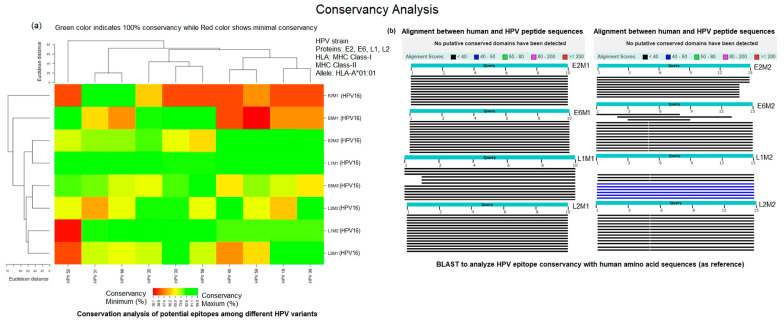
Conservation analysis (**a**) potential epitopes of HPV-16 strain among different variants of HPV sub-types (**b**) sequence alignment of potential epitopes against human proteome as reference to analyze conserved domains.

We predicted T-cell epitopes of E2, E6, L1, and L2 proteins for MHC class I and II molecules. 8-substantial epitopes for each class were found containing 10 amino acid residues of MHC class I and 15-mer residues for class II (Table 1). These T-cell epitopes have the potential to target the maximum number of alleles indicating conservation in the entire populations. Such multi-allelic epitope-based vaccine candidates have more spectral features compared to those candidates that target only one type of allele. The conservancy analysis demonstrated the broad-spectrum activity of these MHC class I and II epitopes against different virulent strains of HPV. Three epitopes, STDLRDHIDY, KISEYRHYCY, and LTSRRTGIRY, indicated 100% conservation, whereas the remaining epitopes showed greater than 70% conservation. BLAST alignment analysis using human proteome as reference sequences presented that there are no putative conserved domains are available in these potential epitopes (Figure 1).

### 2.2. Validation of Potential Vaccine Candidates

**Figure 2:**
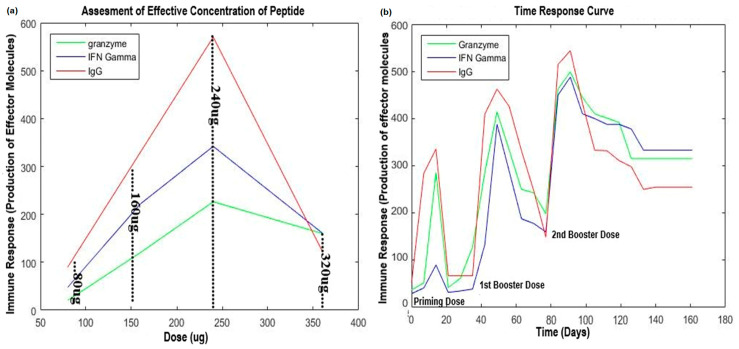
Assessment of effective concentration of potential epitopes (**a**) and time response curve (**b**).

The efficacy of these potential epitopes was investigated by in vivo animal studies with and without adjuvant at optimized concentrations. Significant immune response (time-response curve) was observed at 240 µg/500 µL (Figure 2). We analyzed immune responses of rats to synthetic peptides 3 weeks post immunization. The rats were monitored on daily basis for these three weeks to observe side effects of the administered doses.

### 2.3. Hematological Assays

**Figure 3:**
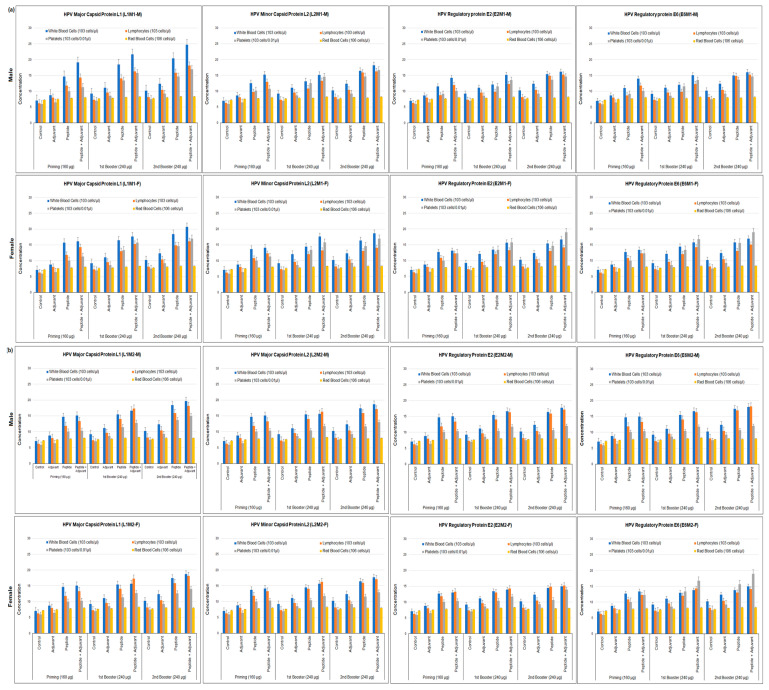
Hematological assays show the cellular count of white blood cells (WBCs), lymphocytes (LYM), platelets (PLT) and red blood cells (RBCs) in serum samples of male and female rats immunized with (**a**) MHC class-I (**b**) MHC Class-II specific monovalent antigenic peptides (individual peptides) emulsified with and without adjuvant at primary, first and second booster doses. All data were compared to control group.

Hematological assays indicate the primary response to the peptides. The blood components of rats were analyzed after immunization to evaluate the efficiency of epitopes, tested with or without adjuvant. The control rats were used to compare with sampled animals and both were kept as same conditions. A substantial increase in the count of different blood Cells was observed when treated with predicted antigenic peptides. At second booster dose of 240 µg, an increased numbers of white blood cells (WBCs), lymphocytes (LYM), platelets (PLT) and RBCs were observed in the samples compared to priming and first booster doses and control. In our analysis, we studied the gender-wise immune response and observed that the epitope of L1M1 (viral major capsid protein) in complex with CFA showed a substantial level of WBCs in male rat (24.68 × 103 cells/μL) compared to female rat samples at the second booster dose. However, the antigenic peptides L2M1 (18.16–18.68) and E2M1 (16.17–16.67) revealed almost the same level of immune response in female and male rats at this concentration. We observed an insignificant level with independent injection of CFA (Figure 3a). Similarly, at the second booster doses (240 µg) of MHC class II peptides, a significant number of WBCs, LYM, and platelets were observed in both male and female rats. L1M2 induced a comparatively increased level of production of WBCs and LYM compared to L2M2, E2M2, and E6M2 (Figure 3b).

### 2.4. IgG ELISA Assay

**Figure 4:**
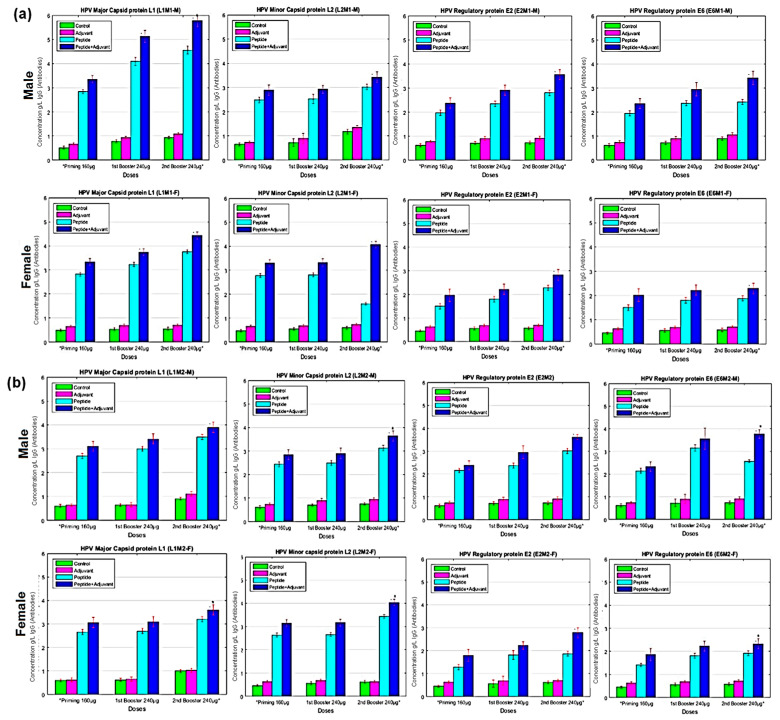
IgG analysis of (**a**) MHC Class-I (**b**) MHC Class-II, antigenic peptides measured by ELISA assays. Blood samples of experimental male and female rats immunized with potential epitopes (individual peptides) emulsified with and without adjuvant at primary, first and second booster doses. The titer rate of IgG antibodies is given in g/L. All data were compared to control group. Bars represent ‘mean; which is signified (*) with *p*-value (<0.01) and standard deviation (SD).

The level of IgG was tested at 3 week post immunization in the blood serum of the immunized rats to analyze the T-cell-dependent B-lymphocyte activation. Second booster doses (240 µg/500 µL) of MHC class I specific L1M1, L2M1, E2M1, and E6M1 elicited a considerable immunological response compared to priming doses. We observed a substantial concentration of IgG antibodies in male rats (5.77 g/L) at the second booster of L1MI-CFA compared to female rats (4.42 g/L). The IgG concentration was lower in rats treated with E6MI (3.42 g/L). We found a trivial response in the case of independent doses of CFA. Compared to individual peptide, adjuvant, and control groups of male rats, MHC class II specific peptides in combination with CFA produced an optimal concentration of IgG antibodies. Similarly, L1M2 elicited a significant immune response compared to L2M2, E2M2, and E6M2 (Figure 4).

### 2.5. IFN-γ ELISA Assay

**Figure 5:**
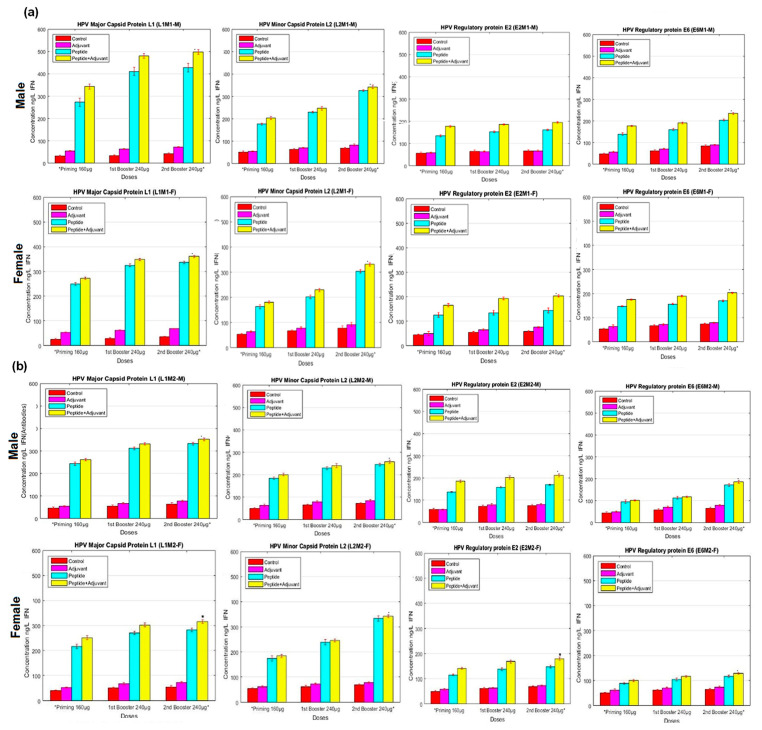
IFN-γ analysis of (**a**) MHC Class-I (**b**) MHC Class-II, antigenic peptides measured by ELISA assays. Blood samples of experimental male and female rats immunized with potential epitopes (individual peptides) emulsified with and without adjuvant at primary, first and second booster doses. The titer rate of IFN-γ is given in ng/L. All data were compared to control group. Bars represent ‘mean; which is signified (*) with *p*-value (<0.01) and standard deviation (SD).

Interferon-gamma concentration was analyzed in blood sera of immunized rats by MHC-I specific peptides (L1M1, L2M1, E2M1, and E6M1) used in combination with CFA. We found a substantial concentration of IFN-γ compared to the control at the second booster dose of LIM1 peptide used with CFA in both male and female rats. The concentration of IFN-γ was lower in rats immunized with individual peptides. We observed that MHC-II specific peptides (L1M2, L2M2, E2M2, and E6M2) stimulated less IFN production compared to MHC-I peptides. However, among these, MHC-II specific peptides, either used individually or peptides with CFA produced considerable immune response in terms of IFN production. At this dose, peptide used with CFA, the L1M2 peptide showed the increased concentration of IFN compared to the control (Figure 5).

### 2.6. Granzyme B Assay

**Figure 6:**
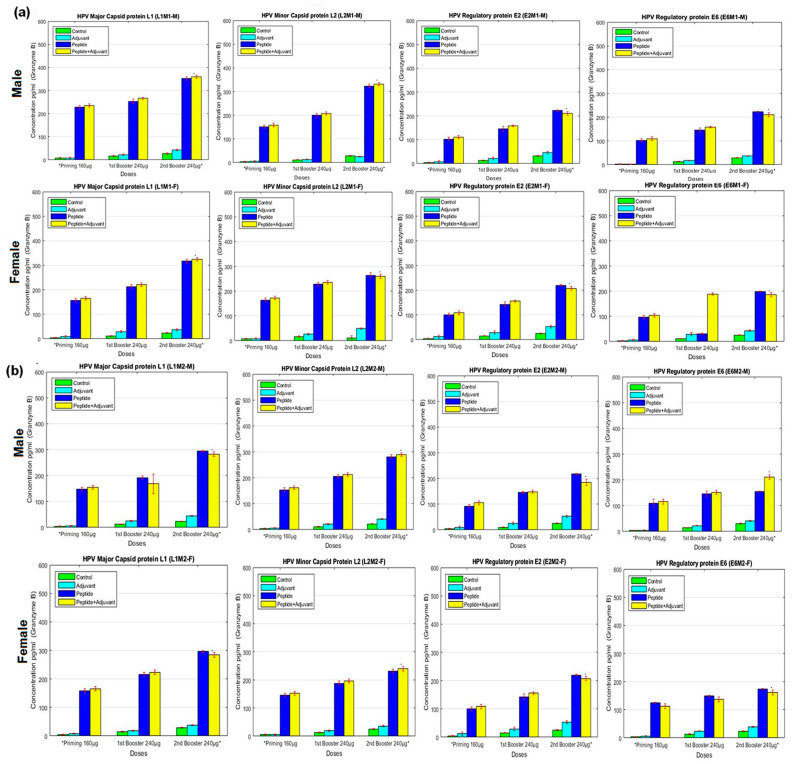
Granzyme B analysis of (**a**) MHC Class-I (**b**) MHC Class-II antigenic peptides measured by ELISA assays. Blood samples of experimental male and female rats immunized with potential epitopes (individual peptides) emulsified with and without adjuvant at primary, first and second booster doses. The titer rate of Granzyme B is given in pg/mL. All data were compared to control group. Bars represent ‘mean; which is signified (*) with *p*-value (<0.01) and standard deviation (SD).

The serum samples of the immunized rats were analyzed for granzyme B production (pg/mL) in response to the administered synthetic peptides. We observed lower concentrations of granzyme in male and female rats immunized with individual peptides and high in L1M1 used with CFA at the second booster dose. It was found that MHC-I specific peptides showed significant immune response compared to MHC-II specific peptides. However, compared to individual peptide, adjuvant, and control groups of female rats, all MHC-II specific peptides produced increased concentrations of granzyme when the peptides were used with CFA. Among L2M2, E2M2, and E6M2, it was observed that L1M2 elicited a high concentration of granzyme (Figure 6).

### 2.7. CD4+ and CD8+ Cell Count

**Figure 7:**
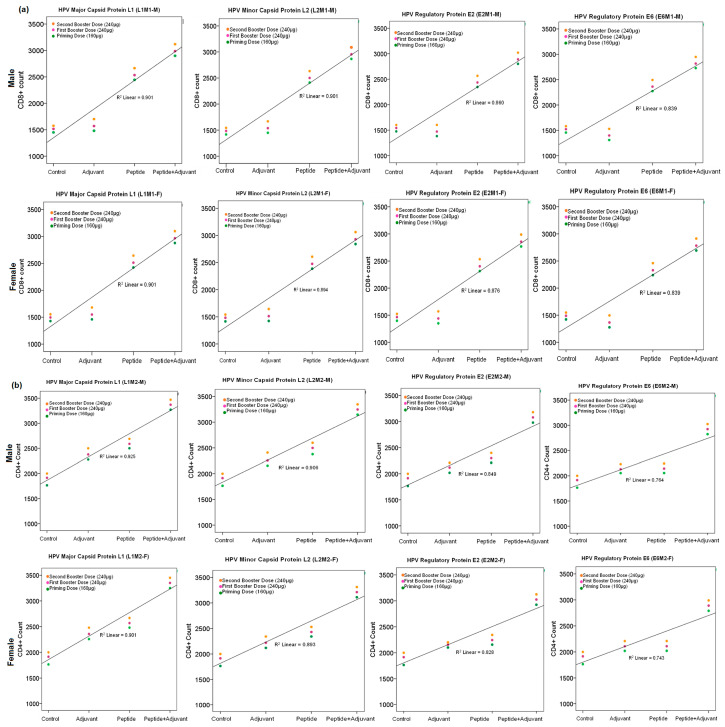
Line graphs expressing CD4+ and CD8+ T cells derived from rats immunized with (**a**) MHC class-I and (**b**) Class-II potential monovalent epitopes (individual peptides) with and without adjuvant at primary, first and second booster doses (R^2^ > 0.7). All data were compared to control group.

Because trypan blue can form complexes with proteins in both the cytoplasm and the plasma membrane, a variety of trypan blue concentrations and incubation times are used to find the best experimental condition for distinguishing between live cells with trypan blue-protein interactions only on the cell surface and dead cells with trypan blue-protein complexes in both the cytoplasm and the cell surface. The cell count analysis showed that the magnitude of CD8+ and CD4+ T cell responses seems directly related to the peptide dose. Individual peptides and peptides with CFA showed an elevated level of CD4+ and CD8+ cells compared to the control and adjuvant groups at the second booster doses. In male and female rats, CD8+ cell count of major (L1) and minor capsid peptide (L2) used with CFA is 3000 and 3118, respectively, and similarly the CD4+ cell count is 3369 and 3484 respectively compared to control (R^2^ > 70%) (Figure 7).

## 3. Discussion

Human papilloma virus is responsible for cervical, head and neck, and other anogenital cancers. It accounts for more than 239,000 deaths around the world annually [16]. Current study predicts potential HPV peptide antigens. Our predicted antigenic peptides induce protective immunity. The strategy provides a timely analysis for vaccine development against different infectious agents. There are recent reports that peptide-based vaccines have been developed for SARS-CoV-2 [17], tuberculosis [18], respiratory syncytial virus (RSV) [19], Chikungunya virus [20], *Streptococcus pneumoniae* [21] and *Neisseria meningitides* [22].

The conventional treatments are not sufficient in treating cancers caused by HPV because of the chance for reoccurrence of cancer, cost, expertise, access, and side effects. Gardasil and Cervarix are two commercially available vaccines, however, there are safety concerns associated with these vaccines, as both vaccines are ineffective in sexually active females and they are not beneficial for pre-existing lesions. Both vaccines are not recommended for pregnant females. Both vaccines are not cost effective and it has been reported that gardasil is linked to Guillain-Barre syndrome (GBS) [23]. Approved vaccines are recombinant VLPs (Virus-like particles) containing the major capsid protein L1 are costly and not broad spectral vaccines [24]. These vaccines target only the four high-risk HPV subtypes 6, 11, 16, and 18 [25,26]. Furthermore, it is unclear whether the presence of a VLP for one kind of HPV affects the immune response elicited against another [27]. These limitations diverted attention to designing epitope-based vaccines for the prevention of HPV infections and HPV-derived cancers. The current study is complementary but it does not overlap with recent studies on HPV vaccine development. The present work applied reverse vaccinology and machine learning strategies to HPV genome and proteome, including both structural and nonstructural viral proteins to predict antigenic peptides. As in vivo screening of enormous vaccine candidates is resource intensive and time consuming [28]. In contrast to conventional reports our study indicated that nonstructural proteins can also be used for prediction of antigenic peptides. 

HPV vaccines must be included in national immunization programs to combat HPV infection [29] and discovery of new and safer vaccines are needed to cope with this situation. Existing multivalent VLP vaccines are currently being investigated and may give broader protection against the majority of high-risk HPV strains [30]. Therefore, the focus shifted to HPV typing through field testing in conjunction with new HPV vaccine programs for cancer prevention. In addition, the effects of vaccine modulators, manner of delivery, expanded coverage of HPV strains, and effects on men and targeted preadolescents or adults should all be considered when determining the best age for effective vaccination [31]. Studies looking through the PubMed, EMBASE, Cochrane Library, and clinicaltrials.gov databases have suggested strategies for developing vaccines that elicit an immunogenic response to high-risk HPVs [32]. In this study, we predicted the peptide-based potential vaccine candidates and effectiveness to prevent HPV infection. Based on antigenicity and specificity, our findings provide a list of potential peptides for generating an immune response. The integration of system-level high-throughput approaches has permitted us to find antigenic proteins from which we found T-cell epitopes. We used the immunoinformatics approach sequentially and reached the point that epitopes from E2, E6, L1, and L2 HPV proteins are more immunogenic. These epitopes have shown the lowest binding energy and affinity for MHC-I and MHC-II molecules. Of eight identified MHC-I binding antigenic epitopes, four epitopes, i.e., E2-HLA-A*01:01, E6-HLA-A*01:01, L1-HLA-A*01:01 and L2-HLA-A*01:01 were selected based on E-score of binding affinity with target MHC-I protein. Similarly, four epitopes include E2-HLA-DRB1*01:01, E6-HLA-DRB1*01:01, L1-HLA-DRB1*01:01 and L2-HLA-DRB1*01:01 for MHCII were selected. All these 8 epitopes were more than 70% conserved in most of the pathogenic strains of HPV. The assessment of safety and protection of optimal dose of predicted antigenic peptides was performed in Sprague- Dawly rat model. The cellular and humoral responses of Sprague Dawly rats following administration of different concentration of doses were observed carefully. Our trials indicated that hematological factors were under normal range in all groups (control, adjuvant, peptide and peptide plus adjuvant groups) for all MHCI and MHCII peptide trials. It shows the non-toxicity of our peptides vaccine candidates for the blood cells, liver, kidneys and other organs [33]. Immunogenicity of vaccine candidates was observed by evaluation of humoral immune response using IgG, IFNγ and Granzyme B immunoassays. IgG immunoassay is considered a general measure of humoral immunity in response to antigens [34] as IgG plays an important role in inducing protective immunity against pathogens [35]. IFNγ and Granzyme B are important cytokines in conferring immunity indicating the magnitude and durability of the peptide induced cytotoxic T cell responses. In vivo, we used IFNγ and granzyme B ELISA kits quantifying the concentration of IFNγ and granzyme B present in the serum samples obtained from vaccinated rats [36]. A Vaccine is considered effective if an expanding T cell count is observed [37]. Elevated level of IgG concentration, CD8+ and CD4+ cell count and IFN-γ and granzyme B response showed a positive dose–response relationship in case of peptides under analysis. 

It is studied that HPV-peptide activating multiple alleles of MHC provides an effective way to cope with HPV infection as both CD4+ and CD8+ T-cells play an important role in the antiviral immune response as well as B-cell clonal proliferation. The outcomes showed that all eight potential peptides (L1M1, L1M2, L2M1, L2M2, E2M1, E2M2, E6M1 and E6M6) induced significant immunogenic responses in immunized animals as compared to control animals. Hematological assays indicated the increased level of lymphocytes, granulocytes, platelets, and WBCs in the serum of immunized rats. We used to treat and control groups to evaluate IgG antibody, IFN, and granzyme titer by ELISA (Enzyme-linked Immunosorbent assay) [38]. T-cell epitopes are effective for vaccine design and development as they elicit long-term immunity and avoid antigenic drift [39]. Therefore, designing epitope-based vaccines for T lymphocytes using these approaches is cost and time effective promising long-term immunity [40,41]. L1 peptides have produced the best immunogenic response compared to L2, E2 and E6 [42,43,44]. In conclusion, based on the outcomes of immunoassays, our predicted peptides are antigenic in nature and have potential to activate the immune response and generation of effector molecules. The present analysis adds to the preliminary data for the successful design of HPV potential vaccine peptides, and the strategy can also be utilized for designing other epitopes for other infectious diseases.

## 4. Materials and Methods

### 4.1. Ethical Approval

The Bioethics Committee for animals of the Institute of Molecular Biology and Biotechnology, Bahauddin Zakariya University, Multan, Pakistan, approved the experimental procedure with Approval No. IMBB/003/2017. ARRIVE guidelines for animal handling were followed to keep the subject animals in animal house.

### 4.2. Retrieval of Viral Proteomic Data and Prediction of MHC Class I and II Binding Epitopes

Proteomic and genomic sequences of HPV strains were retrieved from Papillomavirus Episteme (PaVE) [45]. Uniprot database and Virus-mPLoc server were used to find subcellular positions of HPV proteins [46]. VaxiJen v2.0 server was used to predict antigenicity of selected proteins [47]. Molecular weight of antigenic protein was calculated using ‘Protein Information Resource’ (PIR) and verified by Expasy tool and Sequence Manipulation Suite (SMS) [48]. MvirDB was used to find proteins that are highly virulent and essential to HPV. Immune Epitope Database (IEDB) tool was used to predict T cell epitopes of selected antigenic proteins for multiple alleles of MHC class-I and II molecules. Epitope conservation analysis was also done by IEDB tool [49]. PEP_FOLD server was used to predict Three-Dimensional (3D) structure of peptides [50]. I-Tasser and Chimera [51,52] tools were used to observe and visualize the epitopic regions and the underlying pattern of amino acids. Protein-Protein Interaction (PPI) analysis is important for the development of therapeutic strategies. Host-pathogen protein interaction helps to reveal therapeutic targets. PPI analysis helps in a prediction of protein functions and their role in inducing the host immunity [53]. We used cytoscape v3.6.0 software to construct PPI network [54]. Molecular Docking and in silico binding affinity of peptides with MHCI and II molecules was calculated by Molecular Operating Environment (MOE) software [14].

**Figure 8:**
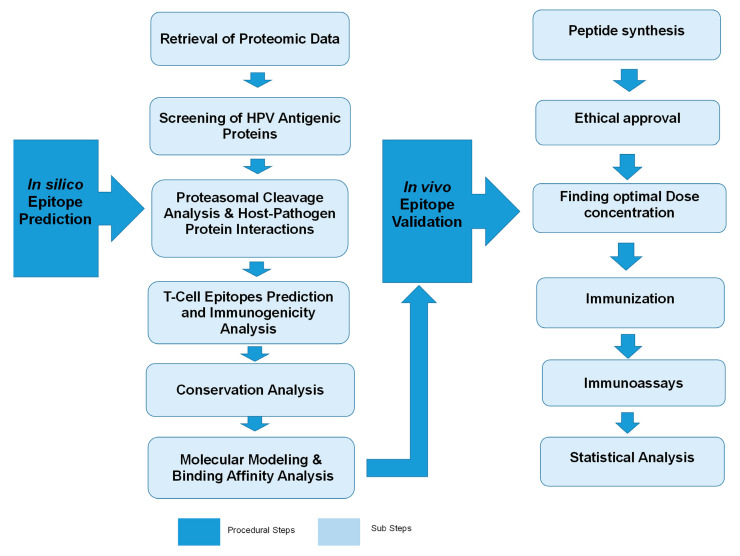
Framework of our methodology for designing potential epitopes of human papilloma virus (HPV).

The framework shown in Figure 8 is applied to predict and design the potential epitopes of HPV using list of tools, servers and databases (Table 2).

### 4.3. Conservation Analysis

The utilization of conserved epitopes in an epitope-based vaccine scenario would be anticipated to offer greater protection across various strains or even species. To evaluate the spectrum and overall effect, we looked at the conservation of our selected epitopes among different variants of HPV. The degree of conservancy of an epitope within a given protein sequences was calculated using the Epitope Conservancy Analysis tool. BLAST analysis at NCBI server was also performed against human proteome in order to exclude HPV epitope conservancy with human amino acid sequences.

### 4.4. Synthesis of Peptides

The predicted T-cell epitopes of MHC class I and II of HPV proteins E2, E6, L1, and L2 with >95% purity was commercially synthesized from SynPeptide Co., Ltd., Shanghai, China.

### 4.5. Dose Optimization and Animal Immunization

Experimental analysis for validation of synthetic peptides and optimization of dose were done using animal models [55]. Sprague Dawley albino rats for preclinical trials were obtained from the breeding unit of the Department of Pharmacology, Faculty of Pharmacy, Bahauddin Zakariya University, Multan, Pakistan. Rats of weight 150 g (male and female rats), 5–6 weeks old were used and divided into four groups (n = 6/group). 160, and 240 µg per 500 µL dose concentrations of E2, E6, L1, and L2 proteins of HPV for MHC-I and MHC-II were given with six weeks interval and we evaluated the optimal dose interval and the number of booster doses required to get the optimal immune response. Eight MHC class I and II specific peptides were tested for their efficacy. These peptides were infused at a similar proportion to these rats individually as well as with the complete Freund’s adjuvant (CFA). One group was injected with peptide alone, the other with peptide and CFA, and the third group was given CFA alone. The animals in fourth group were given nothing as they were taken as controls. A priming dose, the first and the second booster doses were given with the same pattern to each of the four groups to observe the optimal immune response. The rats were kept under observation for a certain period 3 weeks after administration of different doses. Blood samples were taken after 3 weeks post-immunization for hematological and immunoassay. Blood samples were collected by rupturing the vein in the eyes of rats using capillaries of 75 mm length and 1.1–1.2 mm inner diameter [56] (Supplementary Table S1).

### 4.6. Hematological Assays

The levels of total white blood cells (WBCs), lymphocytes (LYM), red blood cells (RBCs) and platelets (PLT) in the blood of immunized rats were analyzed by CBC Coulter (Convergent Technologies GmBH & Co. KG, Coelbe, Germany]. Coulter principle was used for the counting of WBC, LYM, RBC and PLT in the blood. The outcomes were observed in either percentages (%) or 109 cells/Liter [57].

### 4.7. IgG ELISA Assay

Serum samples of each group were analyzed for IgG after three weeks of immunization using ELISA kit as per manufacturer guidelines (Roche, Basel, Switzerland) and microplate reader (Bio-Rad laboratories, Inc., Hercules, CA, USA). Specific ELISA assay was performed using Antigen-Down ELISA Development kit (catalog # 9101, Immunochemistry Technologies, Bloomington, MN, USA) in which potential epitopes were coated in the wells to quantify the IgG. In ELISA-based technique, a specific antigen of animals is coated on wells to record the IgG. The coating reagents (Na_2_CO_3_ buffer 0.95 *w*/*v* of 0.5 M sodium azide at a pH of 9) with 50 µL of capturing antibody in the final concentration of 25 µL/mL were dispensed in wells with appropriate dilution. The microwell plate was incubated for 90 min and after incubation, the solutions were discarded and dried on cellulose paper. 200 µL of washing solution which contains 0.9% *w*/*v* NaCl and 0.1% *v*/*v* Tween-20 was used to wash the wells. 200 µL of 1X blocking reagent (1:10) containing gelatin powder, NaCl, and tris HCl buffer was added and incubated for 90 min. Then the solutions were removed and a 200 µL solution of antibodies extracted from serum at a concentration 25 ng/mL was added to a microtiter plate. Steps of removing the solution and washing were repeated after incubation. The anti-mouse (POD) conjugate solution of 50 µL along with 1ml of blocking reagent was poured into the wells. The wells were incubated and washed. Finally, 50 µL of substrate (2,2′-azino-bis (3-ethylbenzothiazoline-6-sulphonic acid) was added and the fluorescence was recorded at 492 nm to analyze the enzyme-substrate interaction indicating the concentration of IgG in samples (g/L). Mean value ± SD of IgG of control rats was taken as standard [55].

### 4.8. IFN-γ ELISA Assay

Interferon gamma (IFN-γ) concentration in rat serum was quantified by using Rat Interferon Gamma ELISA kit of BT Lab (Catalogue # E0103Ra, KorianBiotech Co., LTD, Birmingham, England). All chemicals were brought to room temperature before use. 50 µL standard dilutions were added into the respective wells. 40 µL of serum samples were added into the sample wells and 10 µL of anti-IFN-γ antibodies were added into only sample wells. Streptavidin-hrp (50 µL) was added to the sample and standard wells. We gently mixed all components of wells covered the plate and kept for incubation at 370 C for 1 h. After incubation, we removed the sealer and washed the wells by adding 350 µL wash buffer (1×) for 30 to 60 s. Following washing, 50 µL substrate A and then 50 µL substrate B was added into each well and incubated for 10 min at 370 C in the dark area. To stop the reaction, 50 µL stop solution was added to each well: the blue color of the reaction mixture was changed into yellow color immediately. The absorbance of each well was measured at 450 nm by BIO-RAD (xMarkTM) plate reader within 10 min after adding the stop solution. Results were obtained in ng/L of serum samples when compared to standard solutions of different concentrations [58].

### 4.9. Granzyme B Assay

Blood of immunized rats was analyzed for granzyme B concentration. Serum was separated from whole blood by using a gel clot vial. ELISA was performed by using Granzyme B Platinum ELISA kit (Thermo Fisher Scientific, Catalog # BMS2027, Waltham, MA, United States) according to the manufacturer’s guidelines. First step was washing of the microwell plate twice by using 400 µL washing buffer (PBS with 1% Tween-20). Wells were dried after 10–15 s of washing by tapping a paper towel. Microwell plate was used immediately after washing and drying. 75 µL of dilution buffer was added in duplicate in sample wells (A1/A2) and 100 µL to standard wells (B1/2, G1/2) in duplicate. Wells A1/2 were then filled with 75 µL of the prepared standard solution of granzyme-B. Solutions of the wells were mixed carefully so that the base of the wells must not get scratched. 50 µL solution was transferred into well B1/2. This process was continued until two rows of wells were covered with a standard solution of granzyme B ranging from 480 to 0.7 pg/mL. 50 µL of solution from each well was disposed of. 100 µL of dilution buffer was added to blank wells and 50 µL to sample wells in duplicate. Each sample solution of 50 µL was dispensed into sample wells. Adhesive film was used to cover the wells and incubated for 60 min at 15–25 °C. Plate was placed on a shaker at 400 rpm. Adhesive film was removed after incubation time and all wells were washed with 100 µL of biotin conjugate. After incubation, 100 µL of streptavidin-HRP dilution was added to all wells. Adhesive film was used to cover the plate and an incubation time of 30 min was given at 18–25 °C with shaking at 400 rpm. After that, the wells were washed carefully and 100 µL of TMB as substrate was added into all wells. After incubation of 15 min, color was observed in each well. Upon dark blue appearance, 100 µl of stop solution was added to stop the enzyme reaction. Stop solutions must be distributed equally throughout the well. Microplate reader (BIO-RAD xMarkTM) was used to examine the absorbance of each well at a primary wavelength of 450 nm and a reference wavelength of 620 nm. Concentration of granzyme B was calculated and recorded in pg/mL. The standard was determined by the mean estimation of granzyme B of control rat serum ± SD [59].

### 4.10. CD4+ and CD8+ Cell Count Analysis

CD4+ and CD8+ Cell count analysis helps in the simultaneous detection of multiple cell surface markers in serum samples [60]. Flow cytometry (hemocytometer) was used to count the cells by trypan blue in cell pellet suspension. All samples were equally distributed into two separate vials for antibody staining. An unstained control with identical cell count was used and two sample vials were stained with antibodies of CD8+ and CD4+. Each sample was incubated with 15 µL of CD8+ and CD4+ primary antibodies for 15 min. The phosphate buffer solution at pH 7.4 was used for washing after incubation. Washing was performed at 500 rcf for 5 min at room temperature. The mixture was loaded onto a hemacytometer and examined under microscope. The total number of cells and the number of cells with blue stain were calculated by following formula [61]

$$\mathrm{Total}\;\mathrm{viable}\;\mathrm{cells}\;\left(\%\right)=\frac{\mathrm{total}\;\mathrm{number}\;\mathrm{of}\;\mathrm{viable}\;\mathrm{cells}\;\mathrm{per}\;\mathrm{ml}\;\mathrm{of}\;\mathrm{aliquot}}{\mathrm{total}\;\mathrm{number}\;\mathrm{of}\;\mathrm{cells}\;\mathrm{per}\;\mathrm{ml}\;\mathrm{of}\;\mathrm{aliquot}}\times 100$$

### 4.11. Statistical Analysis

Statistical analysis was performed on the mean values of the results of all replicates with standard error mean (SEM) and standard deviation (SD) [62]. Mean values of immunized groups were compared with the control group to check the level of significance using MATLAB software [63]. To carry out data interpretation, one-way analysis of variance (ANOVA) was calculated by using SPSS software and *p*-value less than 1.0 × 10^−2^ (<0.01) was considered significant ± SED [64].

## 5. Conclusions

HPV peptides are potential vaccine candidates to bind MHC class I and II molecules. The animal models were applied to analyze the efficacy of these epitopes. Design of epitope-based vaccines for HPV through the synthesis of synthetic peptides is efficient, practical, cost, and time effective. It was observed that among the eight peptides, L1 produced the substantial immune response compared to L2, E2, and E6. The revolutionary changes in the field of reverse vaccinology have modified the prophylactic and therapeutic strategies against infectious diseases.

## Figures and Tables

**Table 1:** Predicted MHC class I and II specific epitopes of HPV antigenic proteins and their physiochemical properties.

Protein	Allele	Peptide Code	Peptide Sequence	Percentile Rank	Toxicity Prediction	Hydrop-athicity	Charge	Mol. Weight
L1	HLA-A*01:01	L1M1	CTSICKYPDY	0.3	Non-Toxic	−0.36	0.00	1192.49
	HLA-DRB1*01:01	L1M2	NIYYHAGTSRLLAVG	0.13	Non-Toxic	0.25	1.50	1635.8
L2	HLA-A*01:01	L2M1	LTSRRTGIRY	0.2	Non-Toxic	−0.91	3.00	1222.55
	HLA-DRB1*01:01	L2M2	VDPAFVTTPTKLITY	3.27	Non-Toxic	0.44	0.00	1666.16
E2	HLA-A*01:01	E2M1	STDLRDHIDY	0.15	Non-Toxic	−1.27	−1.50	1234.42
	HLA-DRB1*01:01	E2M2	EMGFKHINHQVVPTL	7.93	Non-Toxic	−0.14	1.00	1750.29
E6	HLA-A*01:01	E6M1	KISEYRHYCY	0.55	Non-Toxic	−0.09	1.50	1361.67
	HLA-DRB1*01:01	E6M2	RHYCYSLYGTTLEQQ	2.91	Non-Toxic	−0.97	0.50	1862.27

* Significantly expressed alleles.

**Table 2:** List of tools, databases and software used in this study.

Sr. No.	Name	Use	Links
1	Papillomavirus Episteme	Retrieval of HPV Genome	https://pave.niaid.nih.gov/#home (accessed on 5 April 2018)
2	Virus-mPLoc	Subcellular Localization	http://www.csbio.sjtu.edu.cn/bioinf/virus-multi/ (accessed on 15 January 2018)
3	MvirDB	Virulence Factor Prediction	http://mvirdb.llnl.gov/ (accessed on 16 January 2018)
4	Vaxijen server	Antigenicity Prediction	http://www.jenner.ac.uk/VaxiJen (accessed on 3 February 2018)
5	Immune Epitope Database	Epitopes Prediction	http://tools.iedb.org/main/ (accessed on 7 February 2019)
6	Protein information resource	Mol. Weight Prediction	http://pir.georgetown.edu/cgi-bin/comp_mw.pl (accessed on 8 February 2018)
7	Immune Epitope Database	MHC-peptide binding	http://tools.iedb.org/main/ (accessed on 1 March 2019)
8	Immune Epitope Database	Epitope Conservation	http://tools.iedb.org/main/analysis-tools/ (accessed on 5 March 2020)
9	PEP-FOLD	Peptide modeling	http://bioserv.rpbs.univ-paris-diderot.fr/PEP-FOLD/ (accessed on 15 March 2019)
10	Uniprot	Protein Information	http://www.uniprot.org/ (accessed on 7 January 2018)
11	NCBI	Genomic/Proteomic data	https://www.ncbi.nlm.nih.gov/ (accessed on 2 January 2018)
12	MOE	Docking	https://www.chemcomp.com (accessed on 7 August 2019)
13	TASSER	Polylinker Design	https://zhanglab.ccmb.med.umich.edu/I-TASSER/ (accessed on 1 September 2020)

## Data Availability

Data in Supplementary Information File.

## Supplementary Materials

The following supporting information can be downloaded at: https://www.mdpi.com/article/10.3390/molecules28041687/s1. Table S1, Dose regimen of potential monovalent (individual) peptides.
