# Supplementary material for: Experimental Validation of MHC Class I and II Peptide-Based Potential Vaccine Candidates for Human Papilloma Virus Using Sprague-Dawly Models
Source: Molecules. 2023 Feb 10;28(4):1687. doi: 10.3390/molecules28041687 (PMC9968051; doi:10.3390/molecules28041687)
Supplement: Supplementary file 1 [file molecules-28-01687-s001.zip › molecules-2090748-supplementary.pdf]

Supplementary Table 1. Dose regimen of potential monovalent (individual) peptides

Epitope	Gender	Sub groups	No. of rats	Priming dose	Duration	1st Booster dose	Duration	2nd Booster dose
L1M1/L1M2/ L2M1/L2M2/ E2M1/E2M2/ E6M1/E6M2	Male	Control	6	no substance given	6 weeks	no substance given	6 weeks	no substance given
		Adjuvant	6	0.01mg/500µl		0.01mg/500µl		0.01mg/500µl
		Peptide	6	160µg/500µl		240µg/500µl		240µg/500µl
		Peptide +Adjuvant	6	160µg+0.01mg/500µl		240µg+0.01mg/500ul		240µg+0.01mg/500µl
	Female	Control	6	no substance given		no substance given		no substance given
		Adjuvant	6	0.01mg/500µl		0.01mg/500µl		0.01mg/500µl
		Peptide	6	160µg/500µl		240ug/500µl		240µg/500µl
		Peptide +Adjuvant	6	160µg+0.01mg/500µl		240µg+0.01mg/500µl		240µg+0.01mg/500µl
